# The Relationship between Seminal Melatonin with Sperm Parameters, DNA Fragmentation and Nuclear Maturity in Intra-Cytoplasmic Sperm Injection Candidates

**DOI:** 10.22074/cellj.2015.15

**Published:** 2015-10-07

**Authors:** Mina Sharbatoghli, Mojtaba Rezazadeh Valojerdi, Mohammad Hadi Bahadori, Reza Salman Yazdi, Leila Rashki Ghaleno

**Affiliations:** 1 Department of Embryology, Reproductive Biomedicine Research Center, Royan Institute for Reproductive Biomedicine, ACECR, Tehran, Iran; 2 Department of Anatomy, Faculty of Medical Science, Tarbiat Modares University, Tehran, Iran; 3 Department of Anatomy, Faculty of Medical Science, Guilan University of Medical Sciences, Rasht, Iran; 4 Department of Andrology, Reproductive Biomedicine Rersearch Center, Royan Institute for Reproductive Biomedicine, ACECR, Tehran, Iran

**Keywords:** Melatonin, DNA Fragmentation, Antioxidant, Sperm Maturation

## Abstract

**Objective:**

Melatonin, the chief secretory product of the pineal gland, regulates dynamic physiological adaptations that occur in seasonally breeding mammals as a response to changes in daylight hours. Because of the presence of melatonin in semen and the mem- brane melatonin receptor in spermatozoa, the impact of melatonin on the regulation of male infertility is still questionable. The aim of this study was to determine the effects of endogenous melatonin on human semen parameters (sperm concentration, motility and normal morphology), DNA fragmentation (DF) and nuclear maturity.

**Materials and Methods:**

In this clinical prospective study, semen samples from 75 infer- tile men were routinely analyzed and assessed for melatonin and total antioxidant capac- ity (TAC) levels using the enzyme-linked immunosorbent assay (ELISA) and colorimetric assay kits, respectively. DF was examined by the sperm chromatin dispersion (SCD) test. Acidic aniline blue staining was used to detect chromatin defects in the sperm nuclei.

**Results:**

There was no significant correlation between seminal plasma melatonin and TAC with sperm parameters and nuclear maturity. However, we observed a positive significant correlation between DF and melatonin level (r=0.273, P<0.05).

**Conclusion:**

Melatonin in seminal plasma is positively correlated with damaged sperm DNA of infertile patients. The mechanism of this phenomenon needs further study.

## Introduction

Melatonin is a hormone secreted by the pineal gland in the brain. It is involved in the regulation of other hormones and in various physiological functions such as coordination of circadian rhythms, sleep regulation, immune function, tumor-growth inhibition, blood-pressure regulation and free-radical scavenging (1). Many studies have confirmed the site of melatonin receptors on the reproductive system (2). Moreover, melatonin controls the timing for releasing hormones in female reproductive system (3). The influence of melatonin on the neuroendocrine-reproductive axis of a variety of experimental animals well documented indicates that this hormone is postulated to play an antigonadotrophic role in the mammalian reproductive system (4). Bornman et al. (3) reported the presence of melatonin in human seminal plasma; however, they observed no influence on sperm motility. van Vuuren et al. (5) found melatonin receptors on human spermatozoa and proposed that melatonin maybe involved in modulating sperm activity. Some reports also indicated that long term administration of melatonin in healthy men could reduce the semen quality (6). Melatonin has been shown to be an effective antioxidant and the most potent physiological scavenger of hydroxyl radicals (7). Recent studies have shown that melatonin is able to protect human spermatozoa from apoptosis and DNA fragmentation (DF) induced by reactive oxygen species (ROS) (8, 9). In addition, it can improve the percentage of motile and progressively motile human spermatozoa (10). In semen from Mithun, melatonin has been shown to develop sperm with DNA integrity, viability and intact plasma membrane (11). Despite this evidence, the relationship between melatonin and male fertility remain ambiguous and controversial. Therefore, this study attempted to assess the relationship between endogenous melatonin in ejaculation with semen parameters and DF in infertile men who underwent assisted reproductive technology (ART).

## Materials and Methods

### Semen source and preparation

This clinical prospective study was approved by the local Ethics Committee at Royan Institute, Tehran, Iran. Written consent for the use of the spermatozoa for research was obtained from 75 patients undergoing intra-cytoplasmic sperm injection (ICSI) according to guidelines established for research on human subjects. A total of 75 semen samples were collected into sterile plastic containers followed by at least 48 hours of sexual abstinence. After 30 minutes of liquefaction at room temperature, semen samples were analyzed for sperm concentration and motility by alight microscopy. Normal semen analysis was performed according to World Health Organization (WHO) guidelines (1993). The percentage of morphologically abnormal spermatozoa was evaluated by Papanicolaou staining (Bahar Medical Laboratory Bldg., Tehran 14146, Iran). The raw semen specimens were centrifuged at 200 g for 5 minutes, seminal plasma were separated and frozen at -70˚C in an ultra-low temperature freezer until further use. We resuspended 10×10^6^ sperm from the pellet in 0.5 ml of Ham’s F10 medium (Sigma- Aldrich, USA) which was used for evaluation of DNA damage and nuclear maturity. Frozen seminal plasma was thawed by placing the vials in an incubator at 37˚C for 20 minutes followed by immediate assessment of the samples for antioxidant capacity and the melatonin assay.

### Measurement of total antioxidant capacity

Total antioxidant capacity (TAC) assessment was performed by colorimetric assay. We added 20 μL of seminal plasma to 1 mL of the reconstituted chromogen (Sigma, USA), 2, 2’-azino-bis (3-ethylbenzthiazoline-6-sulfonic acid (ABTS)-metmyoglobin (Sigma, USA) [10 mL-vial with 10 mL of phosphate-buffered saline (PBS, Merck, Jermany)]. The standard was 20 μL of 6-hydroxyl-2, 5, 7, 8-tetramethylchroman- 2-carboxylic acid (Trolox, Sigma, USA) at a concentration of 1.73 mmol/L. In this method, 20 mL of deionized water was used as the blank. Chromogen (1 mL) was added to the standard, blank, and sample. Initial absorbance (A1) was measured at 600 nm at 37˚C with a spectrophotometer (CECIL 7250, Bio Aquarius, UK). We added 200 μL of H_2_O_2_ (250 mmol/L) to all tubes (standard, blank, and sample) and a second absorbance (A2) was measured exactly after 3 minutes. The difference between A2 and A1 (DA) was calculated. The TAC concentration of the sample, in terms of Trolox equivalents, was then calculated by the following formula: TAC=concentration of the standard×(DA blank-DA sample)/(DA blank-DA standard). The results were expressed as μM of Trolox equivalents (12).

### Melatonin assay

Seminal plasma melatonin was assessed by an enzyme-linked immunosorbent assay (ELISA) kit (Melatonin Elisa, IBL Hamburg, Germany) and ELISA reader (Awareness, STAT FAX 3200). Briefly, samples were stored at -20˚C until biochemical analyses. All samples, standards and controls, were extracted according to the manufacturer’s instructions. Then each extracted standard and control added into the respective wells. After incubation and washing, an enzyme conjugate was added to each well. After further incubation and washing, p-nitrophenyl phosphate (PNPP) substrate solution was added to all wells followed by a PNPP stop solution to halt the substrate reaction. Subsequently, we measured optical density with a photometer at 405 nm.

### Determination of DNA fragmentation

**Fig.1:**
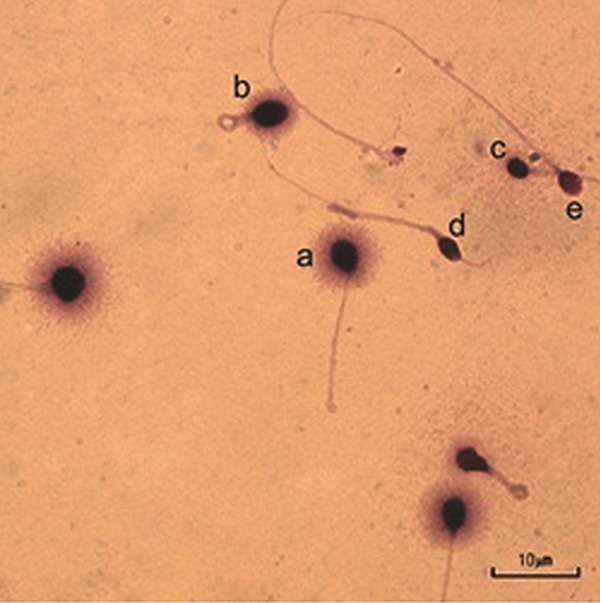
Sperm chromatin dispersion (SCD) test sperm with different size halos. Sperm show: a; Nuclei with large size halo, b; Nuclei with medium size halo that was considered with non-fragmented DNA, c; Nuclei with small size halo, d; Without halo and e; Without a halo and degraded which was considered sperm with fragmented DNA (scale bar=10 μm).

The sperm chromatin dispersion (SCD) test was used to detect DF according to a method reported by Khosravi et al. (13). In brief, an aliquot of a semen sample was diluted to 10 million/ ml in PBS. The suspensions were mixed with 1% low-melting-point aqueous agarose (Fermentas, Canada). Then 50 μL aliquots of the mixture were pipetted onto a glass slide precoated with 0.65% standard agarose (M7730, Cinagen, Iran), and left to solidify at 4˚C for 4 minutes by covering using a coverslip (24-60 mm). Cover slips were carefully removed and slides were immediately immersed horizontally in a tray with freshly prepared acid denaturation solution (0.08N HCl, Merck, Germany) for 7 minutes at 22˚C in the dark. Then the slides were horizontally immersed in 25 ml of the lysing solution, including Tris-Hcl (Merck, Germany) 0.4 M, NaCl 2M, Ethylenediaminetetraacetic acid (EDTA, Merck, Germany) 0.05 M, M-ethanol (Merck, Germany), and sodium dodecyl sulfate (SDS, Sigma, USA) at PH=7.5 for 25 minutes. After being washed for 5 minutes with distilled water, the slides were dehydrated in ethanol (70, 90 and100%, HamonTeb, Iran) for 2 minutes each and then air-dried. Slides were covered with a mix of Wright’s staining solution (Merck, Germany) and PBS (1:1) for 5-10 minutes. Slides were washed in tap water and allowed to dry. For this study, a minimum of 200 spermatozoa per sample was scored under the ×100 objective of a microscope. According to previous study (14), five SCD patterns have been defined: i. Sperm cells with large halos whose halo width is similar to or higher than the minor diameter of the core, ii. Sperm cells with medium-sized halos: their halo size is between those with high and with very small halo, iii. Sperm cells with very small-sized halos: the halo width is similar or smaller than one-third of the minor diameter of the core, iv. Sperm cells without halos and v. Sperm cells without halos and degraded: similar to iv., but weakly or irregularly stained (Fig .1). For calculation of DF, the sperm with large-to-medium size halo of nuclei were determined as sperm with non fragmentated DNA, whereas nuclei with small size halo, without halo and degraded were determined sperm with fragmented DNA.

### Assessment of chromatin maturity

**Fig.2:**
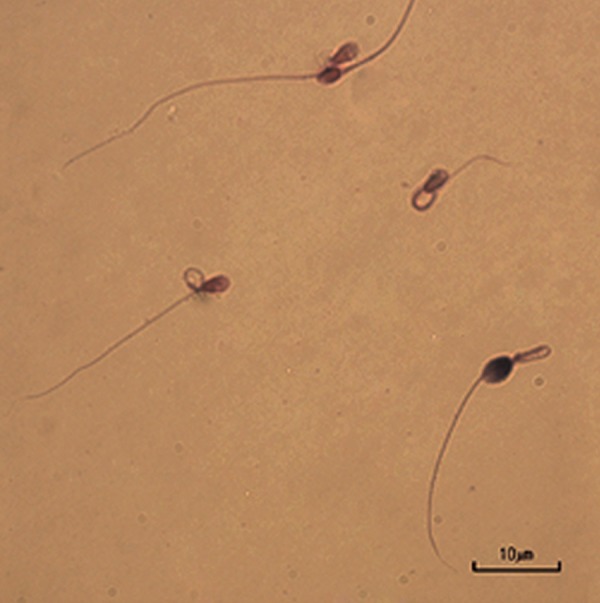
Acidic aniline blue eosin staining. Immature sperm is characterized by nuclear histone proteins stained dark blue, whereas mature sperm with protamine was stained red-pink by the eosin counter stain (scale bar=10 μm).

Sperm chromatin integrity was assessed by aniline blue-eosin staining (Sigma, USA) (15). Briefly, slides were prepared by smearing 5 μl of washed semen sample. The slides were airdried and fixed for 30 minutes in 3% glutaraldehyde (Sigma, USA) in PBS. The smear was dried and stained for 5 minutes in 5% aqueous aniline blue solution (pH=3.5). Sperm heads with excessive histone in chromatin were blue, whereas those with normal histone failed to take up the stain. The percentage of spermatozoa stained with aniline blue was determined by counting 200 spermatozoa per slide under a bright field microscopy (Fig .2).

### Statistical analysis

Pearson correlation test was used to assess correlation of melatonin and TAC with routine sperm parameters and DF. Data analysis was performed using SPSS (SPSS Inc., Chicago, IL, USA) version 20.0 software. P<0.05 was considered significant.

## Results

The minimum, maximum and mean ± standard deviation (SD) of routine semen analyses (concentration, motility and normal morphology), sperm intracellular parameters (DF and nuclear maturity), seminal plasma melatonin and seminal plasma TAC of 75 candidates for ICSI treatment are summarized in table 1. Table 2 shows the correlation of semen melatonin and antioxidants with sperm routine parameters, DF and nuclear maturity. According to the results, we observed no significant correlation between melatonin and antioxidant with semen parameters (concentration, motility and normal morphology) and nuclear maturity. However, there was a significant positive correlation between melatonin and DF (r=0.273, P<0.05).

**Table 1:** Mean of routine classical sperm parameters, melatonin level, total antioxidant capacity level, DNA fragmentation and nuclear maturity in infertile patients who were candidates for ICSI treatment


Sperm parameters	Minimum	Maximum	Mean ± SD

Sperm concentration (×10^6^/ml)	1	160	42.98 ± 35.14
Sperm motility (%)	1	76	29.10 ± 16.06
Sperm normal morphology (%)	0	46	12.02 ± 8.81
Seminal plasma melatonin (pg/ml)	1.80	78	1.99 ± 17.26
Total antioxidant capacity (μM)	0.03	13.70	1.13 ± 1.57
DF (%)	2	54	37.78 ± 24.63
Nuclear maturity (%)	0	89	42.56 ± 20.30


ICSI; Intra-cytoplasmic sperm injection, SD; Standard deviation and DF; DNA fragmentation.

**Table 2:** Correlation between semen melatonin and antioxidant with sperm routine parameters, DF and nuclear maturity in infertile patients who were candidate for ICSI


	Melatonin	TAC

Sperm motility (%)	r=0.071	r=-0.172
	P=0.542	P=0.140
Sperm normal morphology (%)	r=0.048	r=-0.109
	P=0.680	P=0.350
Sperm count (×10^6^/ml)	r=0.112	r=-0.122
	P=0.338	P=0.297
DF (%)	r=0.273	r=0.108
	P=0.018	P=0.357
Nuclear maturity (%)	r=0.135	r=-0.063
	P=0.250	P=0.592
Melatonin (pg/ml)	-	r=0.027
		P=0.817
TAC (μM)	r=0.027	-
	P=0.817	


DF; DNA fragmentation, TAC; Total antioxidant capacity, r; Relation, P; P value and ICSI; Intra-cytoplasmic sperm injection.

## Discussion

Melatonin is as an antioxidant that detoxifies harmful reactants and reduces molecular damage. However, the effect of melatonin on sperm parameters and male infertility are not clear and numerous controversies exist in the reported results.

In the current study, we observed no correlation between the mean of both seminal melatonin and endogenous levels of antioxidant in infertile men. In addition, there was no significant correlation between seminal plasma melatonin and TAC with routine sperm parameters. These results agreed with studies by Luboshitzky et al. (6) and Bornman et al. (3) who measured melatonin level in both blood and seminal plasma. They did not find any correlation between melatonin levels in blood and seminal plasma with routine sperm parameters. In contrast, other researchers have shown a relationship between reduction of sperm motility and low melatonin levels (16). The main reason of these controversial results may be related to number and selection of patients, etiology of infertility, measurement of endogenouse melatonin and its dose in seminal plasma. Accordingly, Espino et al. (9) have shown that 1 mM melatonin has the most inhiibitory action on caspase-9 activation compared with 1 μM. Casao et al. (17) treated seminal plasma obtained from rams with 1 μM, 10 nM and 100 pM melatonin and observed decreased capacitation and phosphatidyleserine (PS) translocation at a concentration of 1 μM and increased short-term capacitation at the 100 pM concentration. Thus, the action of melatonin on spermatozoa function could be dose-dependent. On the other hand, a recent study has shown that the protective effect of melatonin on sperm depends on the interaction between melatonin and its cell surface receptors (9). Therefore, seminal melatonin levels and the number of cell surface receptors are two efficient factors that cause significant correlation in ejaculated spermatozoa. Alterations in these factors possibly generate different results in studies.

Our results revealed a significant positive correlation between melatonin and DF. It was reported that melatonin prevented DNA damage in mouse sperm when treated with diazinon (18). The addition of melatonin preserved DNA integrity in cryopreserved ram spermatozoa (19). It has been demonstrated that ROS is a major reason for DNA damage (20). According to research, melatonin has both antioxidant and anti-apoptotic effects in seminal plasma and the membrane melatonin receptor 1 (MT1) which is involved in regulation of the antiapoptotic effects (9). The positive correlation between melatonin and DF in the current study might have been a good indication of the harmful effects of ROS on sperm membrane melatonin receptors and its feedback control. It has been confirmed that the regulation of melatonin is not compeletly lightdependent and the MT1 receptor is responsible for feedback control release (21).

Accordingly, Espino et al. (9) have recently reported that melatonin’s prevention of H_2_O_2_-induced DF is dependent on both the MT1 receptor and extracellular-signal-regulated kinase (ERK) activation. Therefore, the disruption of melatonin receptors causes enhanced DF by oxidative stress. In addition, the MT1 receptor is responsible for receiving melatonin and delivery feedback control. Possibly its interruption leads to increased melatonin levels in seminal plasma. Moreover, evidence obtained from human pathological studies suggests that melatonin levels has a suppressive effect on pulsatile secretion of gonadotropin-releasing hormone (GnRH) (22) and inhibition effect on leydig cells activity (23). Therefore, in our study, an increase in melatonin might occasionally suppress the hormonal functionality, ultimately leading to a decrease in sperm DNA integrity.

## Conclusion

Although there was no relation between melatonin with seminal plasma, sperm routine parameters and nuclear maturity, it may have an association with sperm DNA damage. More studies are necessary to explain the mechanism of melatonin effect on sperm parameters and its quality.
